# Modification of Physicochemical Properties of Breadfruit Flour Using Different Twin-Screw Extrusion Conditions and Its Application in Soy Protein Gels

**DOI:** 10.3390/foods9081071

**Published:** 2020-08-06

**Authors:** Shiqi Huang, Laura Roman, Mario M. Martinez, Benjamin M. Bohrer

**Affiliations:** 1 Department of Food Science, University of Guelph, Guelph, ON N1G 2W1, Canada; shuang11@uoguelph.ca; 2 School of Engineering, University of Guelph, Guelph, ON N1G 2W1, Canada; lromanri@uoguelph.ca (L.R.); mario.martinez@uoguelph.ca (M.M.M.); 3 Department of Food Science, iFOOD Multidisciplinary Center, Aarhus University, 8200 Aarhus, Denmark; 4 Department of Animal Sciences, The Ohio State University, Columbus, OH 43210, USA

**Keywords:** breadfruit flour, flour modification, extrusion, starch, pasting properties, texture, protein gels, gel stability

## Abstract

The objective was to modify functional properties of breadfruit flours using twin-screw extrusion and test the physicochemical properties of the extruded flours. Extruded breadfruit flours were produced with twin-screw extrusion using different last barrel temperature (80 °C or 120 °C) and feed moisture content (17% or 30%). These conditions resulted in four extruded flours with different mechanical (specific mechanical energy, SME) and thermal (melt temperature) energies. At temperatures below the gelatinization of the native starch (<70 °C), swelling power was increased in all extruded treatments. Solubility was dramatically increased in high-SME extruded flours at all tested temperatures. Water holding capacity was dramatically increased in the low-SME extruded flours. A two-fold higher cold peak viscosity was obtained for low SME-high temperature extruded flour compared with the other extruded flours. Low SME-low temperature extruded flour still exhibited a hot peak viscosity, which occurred earlier than in native flour. Setback was decreased in all extruded flours, especially in high-SME treatments. The incorporation of extruded flours into soy protein gels did not affect cooking loss, while hardness and springiness decreased with the addition of extruded flours. Overall, extrusion of breadfruit flour altered functional flour properties, including water holding capacity and pasting properties, and modified the texture of soy protein gels.

## 1. Introduction

Breadfruit is a high-yielding tropical staple crop grown throughout Oceania, Central Africa, and Central America. Breadfruit is a relatively low-input crop since the tree does not need to be replanted each year and its production lifespan is 50 years or potentially more [1]. Therefore, the breadfruit crop has great potential to positively impact food security in the developing world and has significant economic potential worldwide once the obstacles with developing a global market are overcome [2,3]. Breadfruit flour has shown promise as an alternative flour source with enhanced water retention ability when compared with other more common flour sources (i.e., wheat flour) [4]. Breadfruit flour is a gluten-free flour that has high content of starch [4] and fiber [5], as well as a variety of vitamins and minerals, like vitamin C and potassium [6]. Based on these properties, breadfruit flour has been applied as an ingredient in a variety of processed food products, including pasta [7], instant baby food [8], bread [9,10], cake [11], cookies [12], and meat [13,14]. However, it was reported that inclusion of breadfruit flour in these products should be limited in order to maintain desirable quality characteristics (i.e., high cooking yield, acceptable textural and sensory quality). Some of these shortcomings in product quality stemmed from the high starch gelatinization temperature (peak temperature of approximately 77 °C; Huang et al. [4]) of native breadfruit flour [15,16,17], making its starch incapable of completely swelling. This is likely caused by the entrapment of starch within the fruit tissue matrix when breadfruit flour is added to hydrothermally treated food products that are processed below the starch gelatinization temperature [4].

Chemical, physical, and enzymatic modification have been widely used to improve the functional and physicochemical properties of flours and starches. In recent years, physical modification has garnered attention because of several advantages, such as cost-saving and consumer acceptance (and demand) in terms of natural and clean-labeled ingredients [18,19]. The pre-gelatinization of flour/starch is a typical physical modification technique that results in an improved ability of starch fractions to absorb water and swell at room temperature conditions [20,21]. Extrusion cooking technology is a short time, relatively low-moisture treatment that combines thermal and mechanical energy to produce pre-gelatinized starch while maintaining “clean-label” status (i.e., without the use of chemicals and/or artificial ingredients) [22]. Extrusion promotes starch gelatinization and disrupts the structure of amylose and amylopectin chains, which increases swelling power of starch granules [23,24,25]. The extent of these structural changes is dependent on the many extrusion intensity and processing conditions, such as barrel temperatures, moisture content, feed rate, and screw speed [26,27]. The changes promoted on the constituents of the flours, namely starch gelatinization and protein denaturation, can affect the hydration, thermal, pasting, and rheological properties of extruded/pre-gelatinized flours. Thus, new functional properties are obtained that have potential for several applications in the food industry such as fat-reduction [28,29,30,31,32], reduced-syneresis and staling in sauce and bakery applications [33,34,35,36], and improved rheological properties in meat emulsions due to improved thickening and stabilizing properties [37]. Various physical and chemical modifications of breadfruit starch, including acetylation, oxidation, annealing, heat-moisture treatment, and fermentation, have been studied [38,39,40,41]. In addition, Ma et al. [5] investigated the properties of breadfruit in a variety of extruded and expanded products including dried snacks. However, the functional attributes of breadfruit flours obtained with different extrusion conditions and posterior re-milling of the extruded products remain unexplored. For this reason, breadfruit flour was modified by extrusion in this study to make native breadfruit flour a more suitable ingredient for numerous food applications including both thermally processed and non-thermally processed food products.

Proteins are able to form different gel structures and provide various textural properties in processed food products (i.e., yoghurt, cheese, custards, and sausages). The properties of the gels are affected by the protein source, concentration, pH, ionic environment, processing time, and interactions with other ingredients [42]. Soy proteins usually consist of a well-balanced amino-acid composition and are widely used in many foods; such as bakery products, dairy products, beverages, infant formula, and meat analogues [43]. Combined protein–starch model systems (i.e., soy protein gels formulated with flour) provide a more simplified example for studying the interaction of flours in a protein/starch model system prior to the inclusion of novel ingredients in a more complex food matrix. Thus, this food model system has been previously utilized in research studies to help assess ingredient performance and predict starch–protein interactions [44,45,46]. 

The goal of this study was to modify functional properties of breadfruit flour with twin-screw extrusion technology using different conditions during extrusion. Therefore, breadfruit flours were extruded at different moisture (17% or 30%) and temperature conditions (80 °C or 120 °C in the last barrel) in order to achieve different mechanical and thermal energies. It was hypothesized that extruded flours would have altered viscosity (more viscous at lower temperatures and less viscous at greater temperatures), greater solubility, and improved swelling power when compared with native flour. It was further hypothesized that the extruded flours would have great potential as a texture enhancer and stabilizer in hydrothermal conditions close to the gelatinization temperatures of the native breadfruit flour.

## 2. Materials and Methods

### 2.1. Extrusion of Flour

Native breadfruit flour was provided by Natural Foods International (Apia, Western Samoa). Breadfruit flour was extruded as detailed in Pico et al. [47] with slight modifications. The native breadfruit flour was processed in a Coperion ZSK MV PLUS 27 twin-screw extruder (Ramsey, NJ, USA) with co-rotating, closely intermeshing screws that were 27 mm in diameter with a length to diameter ratio of 24 (L/D). The extruder was equipped with 6 barrel sections. The die head consisted of two circular dies with a diameter of 4 mm each. The temperature of barrel sections 2, 3, 4, and 5 was controlled and remained constant (from the feeding to the die zone) at 40 °C, 60 °C, 80 °C, and 80 °C, respectively. The temperature of the last barrel was controlled and adjusted to either 80 °C or 120 °C. The total moisture content of the product inside the barrel was adjusted to 17% or 30%. These values were based on a mass balance that was performed considering the initial moisture of the solids and then adjusting the mass flow of the water stream accordingly. This resulted in four extrusion treatments (2 × 2 factorial design) varying in specific mechanical energy (SME) and melt temperature (the temperature at which the product comes out of the extruder). The four extruded flours obtained were defined as (1) Low SME-Low Temperature (LS-LT), (2) Low SME-High Temperature (LS-HT), (3) High SME-Low Temperature (HS-LT), and (4) High SME-High Temperature (HS-HT). The main extrusion parameters and full details of the extruded flour treatments are detailed in Table 1. 

Extruded products were dried overnight at room temperature and then ground with a rotor mill (Rotor Beater Mill SR 300, Retsch, Haan, Germany) equipped with a 500 μm mesh screen. Extruded breadfruit flours were stored in air-tight plastic bags at −20 °C until further analyses. 

### 2.2. Characterization of Flours

#### 2.2.1. Proximate Composition

Moisture, lipid, protein, and ash content were determined with the AOAC methods 925.10, 920.85, 992.15, 923.03, respectively [48,49,50,51]. Total starch content was determined according to AOAC method 996.11 [52] following total starch assay kit instructions (Megazyme International Ltd., Bray, Ireland). The samples were washed with aqueous ethanol (80% *v*/*v*) to remove D-glucose and stirred with 2 M potassium hydroxide at approximately 4 °C (ice/water bath) to pre-dissolve resistant starch.

#### 2.2.2. pH

pH values were measured in a homogenate prepared with 2 g of flour sample (both native and extruded flour samples) and 10 mL of distilled water using a benchtop pH meter (AR15 Accumet Research, Thermo Fisher Scientific, Mississauga, ON, Canada) following calibration with buffer solutions of pH 4.0 and pH 7.0.

#### 2.2.3. Water and Oil Holding Capacity

Water and oil holding capacity were measured using similar methodology that was previously reported by Huang et al. [4]. The flour samples (1 g for both native and extruded flour samples) were added to 15 mL of distilled water or 15 mL of refined corn oil for the determination of water holding capacity and oil holding capacity, respectively. Contents were mixed using a vortex mixer for 2 min, followed by centrifugation for 20 min at 6000× *g* on a benchtop centrifuge (Model 21000, IEC International Equipment Company, Needham Heights, MA, USA). The clear supernatant was carefully discarded following centrifugation. Water holding capacity was expressed as grams of water bound by 1 g of dried flour sample. Oil holding capacity was expressed as the weight in grams of oil bound to 1 g of dried flour sample.

#### 2.2.4. Pasting Properties

Pasting properties of the flours (both native and extruded flour samples) were analyzed using a Rapid Visco Analyser 4800 (RVA 4800, Perten Instruments; a PerkinElmer Company, Macquarie Park, Ryde, NSW, Australia) with the maximum holding temperature set at 95 °C. The moisture of flour samples was measured prior to analysis using a moisture analyzer (MA35M-115US, Sartorius Lab Instruments GmbH & Co. KG, Goettingen, Germany) in order to obtain the correct flour sample weight and the amount of water required for the test. The flour suspension (28.5 g total weight) was poured into the RVA canister. Subsequently, the samples were subjected to a heating-cooling cycle in which temperature was initially held at 25 °C for 6 min, increased to 95 °C with a heating rate set to 14.0 °C/min, maintained at 95 °C for 6.5 min, decreased to 25 °C with a cooling rate set to 14.0 °C/min, and finally maintained at 25 °C for 5 min. 

The following pasting parameters were calculated from the pasting curves—cold peak viscosity as the maximum viscosity of the flour slurry at room temperature (25 °C); hot peak viscosity as the maximum viscosity during the heating step; breakdown as the difference between minimum viscosity upon cooling and hot peak viscosity; final viscosity as the viscosity at the end of the test, which indicated the ability of the flour to form a viscous paste or gel after cooking and cooling; setback obtained as the difference between minimum viscosity upon cooling and final viscosity; and peak time was estimated as the time at which hot peak viscosity was reached.

#### 2.2.5. Scanning Electron Microscopy

Scanning electron microscopy (JCM-5000 Benchtop SEM, Jeol Ltd., Tokyo, Japan) was used for the determination of granule morphology. Flour samples were sprinkled onto double-sided adhesive tape attached to a circular specimen stub. A uniform layer of sparsely scattered particles was obtained after carefully removing excess powder with a brush. The specimen holder was then transferred to the sample chamber and the flour samples were observed and photographed at an accelerating potential of 10 kV.

#### 2.2.6. Solubility and Swelling Power

Solubility and swelling power were measured using a similar methodology that was previously reported by Huang et al. [4]. One wt% aqueous suspension of each flour sample (ratio of 0.1 g flour for every 10 mL of water) was prepared in a 500 mL glass flask and was heated at desired temperatures (30, 50, 70, and 90 °C) for 1 h with constant stirring using a magnetic stir bar and a stirring hotplate (Fisherbrand Isotemp stirring hotplate; Thermo Fisher Scientific, Mississauga, ON, Canada). Samples were poured into a weighed centrifuge tube and centrifuged (Model 21000, IEC International Equipment Company, Needham Heights, MA, USA) at 3000× *g* for 10 min. The supernatant was poured into a weighed aluminum dish and evaporated at 100 °C in a drying oven (Fisherbrand Isotemp 180 L drying oven; Thermo Fisher Scientific, Mississauga, ON, Canada) for 24 h. The weight of dry solids and the weight of wet sediments in the centrifuge tube were determined for the calculation of solubility and swelling power, respectively.

Solubility (%) = (the weight of flour dissolved in water/the weight of total flour sample) × 100%(1)

Swelling power (g water/g flour) = the weight of the wet sediments/(the weight of total flour sample × (100%—solubility))(2)

### 2.3. Characterization of Soy Protein Isolate Gels

#### 2.3.1. Preparation of Soy Protein Isolate Gels

Soy protein isolate (MyVegan, The Hut Group, Shepherdsville, KY, USA) and sodium chloride (Hela Spice Canada Inc., Uxbridge, ON, Canada) were sourced from commercial vendors. Water, soy protein isolate, flour (both native and extruded flour samples), and sodium chloride were formulated at consistent levels of 74%, 20%, 4%, and 2%, respectively. The soy protein isolate and flour inclusion levels of gels was kept constant at inclusion levels of 20% and 4%, respectively, to ensure that a self-supporting gel was formed. The soy protein isolate inclusion level of 20% was similar to previous studies [43,53]. The flour inclusion level of 4% was deemed appropriate for the test based on preliminary experiments. Initially, sodium chloride was dissolved in water to create a salt solution. Subsequently, flour was mixed thoroughly with the soy protein isolate. Flour and protein isolate were then added and mixed thoroughly in the salt solution using a spatula until a homogenous batter was generated. 

Thirty grams of uncooked, mixed and prepared protein/flour batter was placed in 50 mL centrifuge tubes with constant agitation to prevent air voids. Samples were placed in a water bath (Haake W26 Fisons, Berlin, Germany) set to 87 °C and cooked to an internal temperature of 85 °C. The samples remained at 85 °C for 25 min. The cooking temperature was kept constant at 85 °C to ensure that a self-supporting gel was formed after cooking. This endpoint cooking temperature was determined based on preliminary experiments.

#### 2.3.2. Cooking Loss

After cooking, test tubes were removed from the water bath and inverted for 16 h at room temperature to release the exudate. The samples were weighed again to determine cooking loss after the release of the exudate.

Cooking loss (%) = (the weight of liquid loss of cooked protein/flour batter (g)/the weight of uncooked protein/flour batter (g)) × 100(3)

#### 2.3.3. Textural Properties

Texture profile analysis (TPA) of protein gel samples was conducted using a texture analyzer (Model TA-XT Icon, Stable Micro Systems, Texture Technologies Corp., South Hamilton, MA, USA) in accordance with methodology described previously by Huang and Bohrer [14]. Protein gels were cooked using the same methodology as described for cooking loss. The cooked protein gels were stored in refrigeration at 4 °C for approximately 24 h before the TPA assay was performed. Prior to TPA, cooked samples were removed from the refrigerator and tempered to room temperature (approximately 21.5 °C). Subsequently, the cooked samples were cut into cylindrical cores (10 mm length and 28 mm diameter) and prepared for TPA. Six cylindrical cores were obtained from each gel sample. The sub-samples were compressed twice (instantaneously) at 25% of their original height with a 101.6 mm diameter cylindrical acrylic probe (TA-40A; Texture Technologies Corporation, South Hamilton, MA, USA) at a constant crosshead speed of 1.5 mm/s. The following TPA parameters were analyzed: hardness (N), gumminess (no units), springiness (%), adhesiveness (g·sec).

#### 2.3.4. Instrumental Color

Instrumental color was measured using guidelines presented by the American Meat Science Association [54]. Instrumental color was determined with a calibrated, handheld Minolta Chroma meter (Konica Minolta Sensing Inc., Osaka, Japan) using illuminant D_65_, a 0° viewing angle, and an 8 mm aperture. The standard CIELAB Color System (L* = lightness; a* = red to green; b* = yellow to blue) was used for the evaluation of instrumental color. The cooked protein gel samples were freshly cut into cylindrical cores (10 mm length and 28 mm diameter) immediately prior to evaluation. Six cylindrical cores were obtained and evaluated from each gel sample.

### 2.4. Statistical Analysis

All laboratory procedures were conducted in three independent replications for each treatment (*n* = 3 for each flour). Each replication was at the minimum conducted in duplicate for each laboratory procedure, and the coefficient of variation thresholds were used to ensure precision and repeatability. Statistical analyses were performed using Statistical Analysis System software (SAS 9.4, SAS Inst. Inc., Cary, NC, USA). The data were analyzed with PROC GLIMMIX of SAS with the fixed effect of flour and the random effect of replication. Least square means were separated using the PDIFF option with a Tukey–Kramer adjustment. Differences were considered statistically different at *p* ≤ 0.05. Pearson correlation coefficients were calculated among parameters using PROC CORR of SAS.

## 3. Results and Discussion

### 3.1. Characterization of Native and Extruded Breadfruit Flours

The SME attained during extrusion and melt temperature in the die section are important parameters as both directly reflect the impact of the extrusion conditions on the structural characteristics of the resulting extruded material [55,56]. In this study, two SME values (74 kJ/kg and 145 kJ/kg) were attained from the adjustment of extrusion conditions, which included moisture content (17% and 30%) and last barrel temperature (80 °C and 120 °C). Specifically, the two extruded flours with low SME values had melt temperatures of 83 °C (LS-LT) and 105 °C (LS-HT), while the other two extruded flours with high SME values had melt temperatures of 100 °C (HS-LT) and 126 °C (HS-HT). As a whole, the combination of these parameters was expected to enable meaningful conclusions to be formed regarding the individual and interactive effects of SME and melt temperature on the physicochemical properties of extruded breadfruit flours.

#### 3.1.1. Proximate Composition

The extruded flours had less (*p* < 0.01) moisture content compared with the native flour. This caused starch, protein, lipid, and ash content to differ (*p* < 0.05) on an as-is basis (Table 2). The magnitudes of difference were generally reduced when composition was expressed on a dry-matter basis. The main compositional component of all the flours was starch (ranging from 66.59% to 72.19%). As expected, the extrusion treatments did not affect (*p* = 0.20) starch content in extruded flours when expressed on a dry matter basis. Extrusion did not change (*p* > 0.05) the protein content (expressed on a dry-matter basis) in LS-LT, HS-LT, and HS-HT extruded flours compared with native flour (protein ranged from 5.41% to 5.53%), but the protein content of LS-HT extruded flour (5.26%) was slightly less than (*p* < 0.05) the native flour (5.54%). The lipid content (expressed on a dry matter basis) of the native flour was 3.51%, which was greater than (*p* < 0.05) all the extruded flours (ranging from 1.74% to 1.99%). This was expected, as detectable lipid content is often reduced following extrusion due to reduced extractability [57]. The formation of amylose–lipid and protein–lipid complexes can lead to less free lipid [58]. Björck and Asp [59] reported that detectable lipid measured with lipid extraction using non-polar solvents was lower in extruded wheat and corn when compared with non-extruded materials. In contrast, ash content was greater (*p* < 0.05) for all extruded flours compared with native flour, which may be due to a better extractability of minerals in the disrupted tissue matrix after extrusion.

#### 3.1.2. pH, Water Holding Capacity, and Oil Holding Capacity

The pH value of native flour was 5.74, while pH significantly increased to a more neutral pH after extrusion (ranging from 6.73 to 7.16). In this sense, breadfruit pulp is rich in ascorbic acid (vitamin C; 5.79 mg/100 g to 22.6 mg/100 g), and this value varies with maturity state [60]. However, ascorbic acid is easily destroyed during thermal processing; thus, the significant reduction in ascorbic acid content following thermal processing was in agreement with previous research [61]. Thus, greater pH after extrusion could be related to the partial loss of acidic components, like ascorbic acid during extrusion.

The water holding capacity of extruded flour in this study ranged from 2.66 g water/g flour to 5.01 g water/g flour. These values were similar to the values for the water absorption index (which ranged from 2.543 to 4.285 g water/g flour) of extruded and expandable breadfruit products reported by Ma et al. [5]. Yet, the water absorption index increased as barrel temperature increased in the previous study conducted by Ma et al. [5]. This did not occur in this study, likely due to the differences in extruder configuration. The water holding capacity was dramatically increased (almost double of native flour, *p* < 0.05) for LS-LT and LS-HT extruded flours, while the water holding capacity was only slightly increased (*p* < 0.05) for HS-LT extruded flour and did not significantly increase for HS-HT extruded flour compared with native flour. The differences in water holding capacity among extruded flours was likely due to the fact that extrusion in the lower SME treatments (LS-LT and LS-HT) was carried out at a greater moisture content (30%). Moisture can operate as a plasticizer during extrusion cooking, which can reduce the degradation of starch and lead to an increased water absorption ability [26]. On the other hand, greater shear (higher SME) may result in more solubilisation of degraded starch, making the material less capable of absorbing water [36], as discussed below. 

Oil holding capacity was dramatically lowered (*p* < 0.05) in all extruded flours when compared with the native flour. Among the extruded flours, oil holding capacity was slightly less (*p* < 0.05) in HS-LT extruded flour compared with the other three extruded flours. Oil holding capacity depends on hydrophobic groups in the material and the ability of the material to maintain oil within its structure [62]. The reduction in oil holding capacity in extruded breadfruit flour may be attributed to fewer lipophilic sites being released during extrusion, which might have been initiated from protein denaturation/unfolding and rearrangement of the hydrophilic and hydrophobic groups. When both protein denaturation and aggregation take place during extrusion, interactions among hydrophobic groups also occur during the formation of aggregates, which would lead to a decrease in the hydrophobicity or number of available hydrophobic sites able to react with oil in the resulting extruded material [63].

#### 3.1.3. Pasting Properties

**Figure 1:**
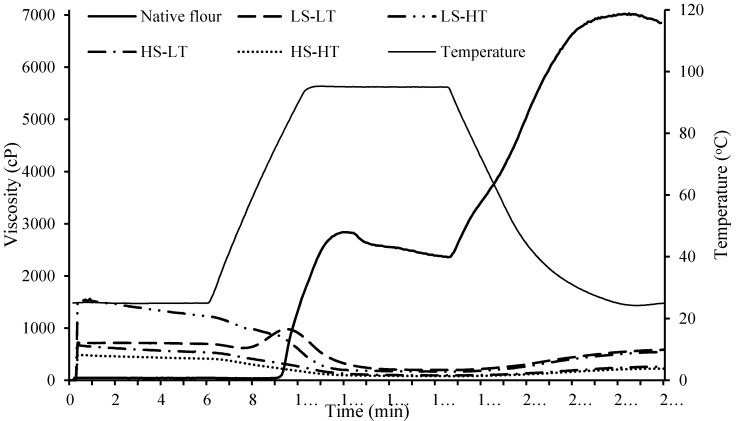
Pasting profile of native and extruded breadfruit flours. Treatments include native breadfruit flour and four extruded breadfruit flours: LS-LT: specific mechanical energy (SME) = 74 kJ/kg, melt temperature = 83 °C; LS-HT: SME = 74 kJ/kg, melt temperature = 105 °C; HS-LT: SME = 145 kJ/kg, melt temperature = 100 °C; HS-HT: SME = 145 kJ/kg, melt temperature = 126 °C.

Pasting viscosity profile and pasting properties of native and extruded breadfruit flours are shown in Figure 1 and Table 3, respectively. When extruded flours were dispersed in water at 25 °C, the cold peak viscosity/initial viscosity observed for all extruded flours suggested the presence of pre-gelatinized starch. LS-HT extruded flour had the greatest (*p* < 0.05) cold peak viscosity and HS-HT extruded flour had the lowest (*p* < 0.05) cold peak viscosity. Lower values of cold peak viscosity suggested more amylopectin was fragmented during extrusion [56]. These results were consistent with the applied SME. There was no hot peak viscosity detected for LS-HT, HS-LT, and HS-HT extruded flours, indicating that these flours were completely gelatinized during extrusion. However, a hot peak viscosity was detected for LS-LT extruded flour, suggesting the starch in this flour was not fully gelatinized. Similar observations for pasting profile with a different degree of starch gelatinization have been reported previously for extruded cereal flours [26,27]. The peak time at which the hot peak viscosity occurred in LS-LT extruded flour was earlier and less pronounced compared with native flour. This could cause significant differences in the technological properties of processed food systems formulated with the two flours during thermal processing and cooking. The native flour had much greater (*p* < 0.05) final viscosity and setback values compared with the extruded flours. During cooling, the extent of the increase in paste viscosity is governed by the tendency of the starch to re-associate by hydrogen bonding, known as retrogradation [64]. The reduction in final viscosity and setback in extruded flours provided evidence of the effect on the amylose chains that were less able to retrograde during the cooling cycle, which is attributed to shear-fragmentation during extrusion [27,56]. This dramatic reduction in the final viscosity of extruded samples compared with corresponding non-extruded samples has been reported previously [24,26,27,56,65]. Overall, breadfruit flour extruded at higher SME levels (HS-LT and HS-HT) displayed lower final viscosity compared with those extruded at lower SME levels (LS-LT and LS-HT), which was likely caused by greater starch fragmentation.

#### 3.1.4. Particle Size and Microscopic Appearance

**Figure 2:**
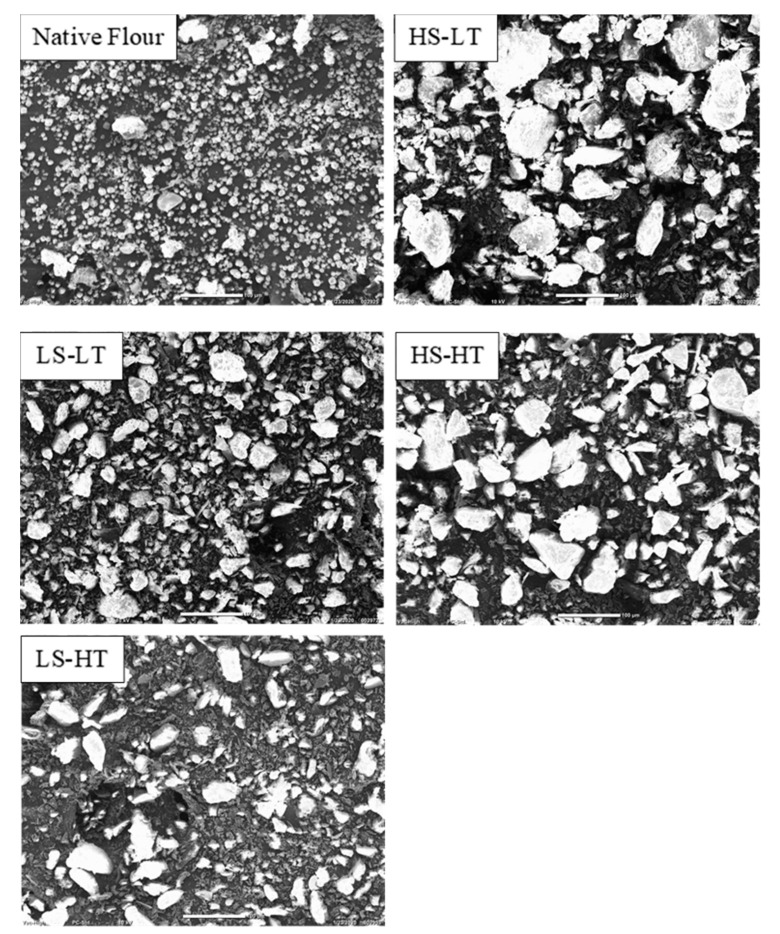
Micrograph images of native and extruded breadfruit flours on scanning electron microscopy. Treatments include native breadfruit flour and four extruded breadfruit flours: LS-LT: SME = 74 kJ/kg, melt temperature = 83 °C; LS-HT: SME = 74 kJ/kg, melt temperature = 105 °C; HS-LT: SME = 145 kJ/kg, melt temperature = 100 °C; HS-HT: SME = 145 kJ/kg, melt temperature = 126 °C.

The typical structure of pre-gelatinized flour was observed in the extruded breadfruit flour samples (Figure 2). Starch granules lost their integrity and amorphous agglomerates were nearly formed. This was in agreement with previous research findings for extruded rice and maize flours [34,66]. Remarkably, the gelatinization of LS-LT extruded flour was not complete as the presence of a hot peak viscosity suggested. However, no rounded or swollen starch granules in this broken structure were visible. Extruded flour particles had a similar size, indicating that the differences in functionalities among extruder flours were not caused by differences in particle size, but rather extrusion conditions.

#### 3.1.5. Solubility and Swelling Power

**Figure 3:**
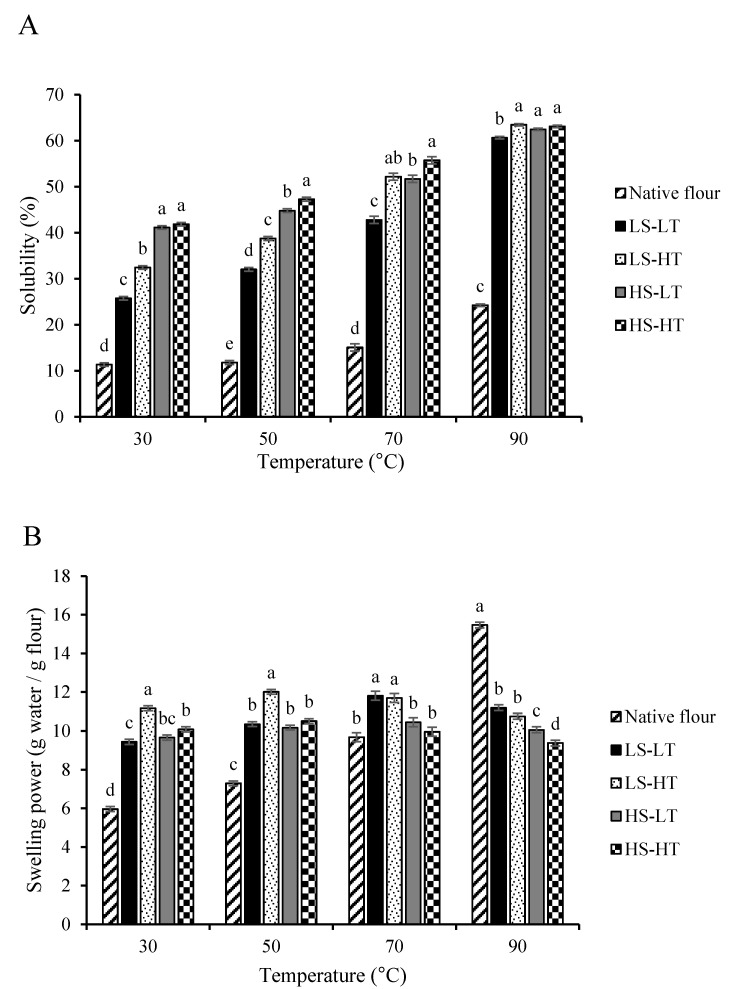
Solubility (**A**) and swelling power (**B**) of native and extruded breadfruit flours. Within each temperature, means without a common superscript differ (*p* ≤ 0.05). Treatments include native breadfruit flour and four extruded breadfruit flours: LS-LT: SME = 74 kJ/kg, melt temperature = 83 °C; LS-HT: SME = 74 kJ/kg, melt temperature = 105 °C; HS-LT: SME = 145 kJ/kg, melt temperature = 100 °C; HS-HT: SME = 145 kJ/kg, melt temperature = 126 °C.

The solubility of both native and extruded flours increased when samples were heated from 30 °C to 90 °C (Figure 3A). At each temperature, the solubility of all the extruded flours was much greater (*p* < 0.05) compared with native flour. At 30 °C, the solubility of extruded flours ranged from 25.76% to 41.84%, while the solubility of the native flour was 11.34%. Likewise, Ma et al. [5] reported that water solubility index of extruded breadfruit products ranged from 54.75% to 65.75% at 20 °C, with water solubility index decreasing as barrel temperature increased. Among extruded flours, the solubility of low SME extruded flours (LS-LT and LS-HT) was generally lower (*p* < 0.05) than that of high SME extruded flours (HS-LT and HS-HT), with significant differences between LS-HT, HS-LT, and HS-HT extruded flours at higher temperatures (70 °C and 90 °C). Low SME extruded flours (LS-LT and LS-HT) were extruded at a greater moisture content compared with high SME extruded flours (HS-LT and HS-HT), which suggests that starch was more degraded in high SME extruded flours due to a greater shear-fragmentation on starch molecules [56]. Interestingly, LS-HT extruded flour had greater solubility compared with LS-LT extruded flour, which indicated that lower temperature and low SME interacted to decrease solubility. This could be related to the fact that the melt temperature of LS-LT extruded flour was 83 °C and below the conclusion temperature of starch gelatinization for breadfruit (88 °C) as reported by Huang et al. [4]. Thus, some of the starch components may still have been trapped inside the remnant swollen granules, and not solubilized within the medium. In fact, differences in solubility between LS-LT and the other extruded flours were minimized at 90 °C, which was well above the gelatinization temperature of breadfruit starch.

The extruded flours were pre-gelatinized and had greater (*p* < 0.05) swelling power at lower temperatures (30 °C and 50 °C) compared with native flour (Figure 3B). Moreover, the swelling power of extruded flours seemed to reach a stable value as temperature increased. The swelling power of pre-gelatinized starches from other sources behaved in a similar fashion. Kittipongpatana [67] reported that the swelling power of pre-gelatinized jackfruit starch was greater than that of native jackfruit starch at lower temperatures and remained stable as temperature increased. Similarly, Zhang et al. [68] reported extruded maize flour had greater swelling power than native maize flour at low temperatures; yet, at elevated temperatures, the swelling power of extruded flour maize flour was lower than native maize flour and it remained stable. 

Conversely, the swelling power for native breadfruit flour increased with increasing temperature, especially from 70 °C to 90 °C, at which point starch gelatinization in breadfruit flours occurs (peak temperature of ~77 °C; Huang et al. [4]). Interestingly, the swelling power of the gelatinized native flour was greater (*p* < 0.05) than the extruded flours at 90 °C (i.e., after complete gelatinization of the starch). Regarding extrusion conditions, LS-HT extruded flour had greater (*p* < 0.05) swelling power at 30 °C and 50 °C compared with the other extruded flours. This was in agreement with the results seen for pasting profile, which showed that LS-HT had greater viscosity compared with the other extruded flour until approximately 65 °C to 70 °C, at which point LS-LT extruded flour reached similar viscosity values. This could imply that LS-HT was more capable of interacting with water compared with the other extruded flours at lower temperatures. However, swelling power was not different between LS-LT and LS-HT extruded flours at 70 °C and 90 °C, yet both were greater (*p* < 0.05) compared with HS-LT and HS-HT extruded flours (i.e., higher SME and possibly higher starch degradation). This was also supported by the RVA results, which showed that LS-LT extruded flour was not fully pre-gelatinized (or the granule structure fully disrupted), as the presence of a small peak swelling at approximately 82 °C suggested. However, this increment in viscosity was less significant, and occurred at an earlier temperature and time than in the native flour whose swelling power was only significantly higher at 90 °C.

### 3.2. Characterization of Breadfruit Flour-Soy Protein Isolate Gels

#### 3.2.1. Cooking Loss

There were no differences (*p* = 0.29) for the cooking loss of protein gels between the flours (Table 4). This indicated that the native flour and the extruded flours had similar water-retention ability in the soy protein gels. Although there were no significant differences between samples, there was a moderate negative correlation (r = −0.49; *p* < 0.10) between the cooking loss of protein gels and the water holding capacity of the flour.

#### 3.2.2. Texture Profile Analysis

The hardness of protein gels was greater (*p* < 0.01) for gels prepared with the native flour compared with gels prepared with the extruded flours. In addition, hardness was greater (*p* < 0.05) for protein gels prepared with low SME extruded flours (LS-LT and LS-HT) compared with gels prepared with high SME extruded flours (HS-LT and HS-HT). The gumminess of protein gels was similar to the values of hardness, as gumminess was lower (*p* < 0.01) in extruded flours compared with native flour. Among the extruded flours, the gumminess of protein gels was greater (*p* < 0.05) for gels prepared with LS-LT extruded flour compared with all other extruded flours, and was greater (*p* < 0.05) for gels prepared with LS-HT extruded flour compared with HS-LT and HS-HT extruded flour. There was no difference (*p* > 0.05) for the springiness of protein gels prepared with low SME extruded flours (LS-LT and LS-HT) compared with native flour. However, springiness was lower (*p* < 0.05) for protein gels prepared with high SME extruded flours (HS-LT and HS-HT) compared with the other flours. The ability of successful gel formation largely depends on the mechanisms of bonding and interactions, such as hydrogen and covalent bonds, and electrostatic and hydrophobic interactions [53,69]. Protein and starch interactions may be primarily based on the unfolding of the protein chains resulting from protein denaturation and the amylose or amylopectin components leaching out from the starch granules during thermal processing (for native sample) or the already solubilized extruded starch in a mostly amorphous state. High SME extruded flours had more mechanical damage and fragmentation of amylopectin molecules, which led to lower viscosity and lower tendency for short-term retrogradation (i.e., setback), two things that may be indicative of the softer gels observed here. In fact, hardness was strongly correlated (r = 0.81, *p* < 0.01) with final viscosity and the setback of the flours. All textural attributes of protein gels were negatively correlated (r ≥ |0.56|, *p* < 0.05) with solubility at 90 °C and positively correlated (r ≥ 0.69 and *p* < 0.01) with swelling power at 90 °C. Moreover, adhesiveness of protein gels was negatively correlated (r = −0.68, *p* < 0.01) with flour water holding capacity and positively correlated (r = 0.86, *p* < 0.01) with setback, which would highlight greater adhesiveness (more negative value) of protein gels prepared with extruded flours. In this sense, starch de-polymerization causes greater stickiness of flours and on the surface of protein gels.

#### 3.2.3. Instrumental Color

Regarding the instrumental color of the gels, there was no difference (*p* > 0.05) for the L* value (lightness) of protein gels prepared with LS-LT, LS-HT, and HS-LT extruded flours compared with protein gels prepared with native flour. Yet, the L* value was greater (*p* < 0.05) for protein gels prepared with HS-HT extruded flour compared with all other treatments. Perhaps more meaningful was that protein gels prepared with extruded flours had a greater (*p* < 0.05) a* value (redness) compared with native flour. Extrusion treatment can cause several heat-sensitive chemical reactions to occur, including the formation of Maillard reaction compounds [57]. Maillard reaction compounds, specifically melanoidins, likely attributed a darker redder color to the protein gels.

## 4. Conclusions

Breadfruit extruded flours varying in cold swelling and retrogradation properties can be obtained by controlling the specific mechanical and thermal energies applied during extrusion treatments. Extrusion treatments did not change the major proximate composition of the flour, namely starch and protein, although it seemed to modify lipid and ash extractability when compared with native flour. The resulting flours had greater pH values after extrusion, indicating that extrusion treatments led to a partial loss of acidic components, such as ascorbic acid. Extrusion conditions that resulted in a lower SME (74 kJ/kg) led to an increased capacity for water absorption and reduced solubility (compared to high SME conditions) as a result of the lower shear-fragmentation of starch molecules, which could be desirable in food applications and result in improvements in processing yields. In fact, a two-fold higher cold peak viscosity was obtained for LS-HT extruded flour compared with the other extruded flours. This flour may prove to be useful in situations where cooking at high temperatures is undesirable and/or in instant food applications. Conversely, low short-term retrogradation properties, as denoted by setback, were obtained for all extruded flours, especially for those subjected to higher SME (175 kJ/kg) during extrusion.

Based on the results of cooking loss and texture in the protein gel modeling systems, it can be concluded that the incorporation of extruded flour can maintain adequate processing yields (i.e., no cooking loss) while adjusting the texture and color of protein/starch food systems. This research is beneficial for the food industry as it provides a characterization of the extruded breadfruit flour and its potential as a gelling agent in protein/starch food systems. Research on the incorporation of extruded breadfruit flour in specific food applications is necessary to further these initiatives.

## Figures and Tables

**Table 1:** Extrusion conditions applied to breadfruit flour.

Extrusion Treatment	Mechanical Energy-Temperature Conditions	Last Barrel Temperature, °C	Total Moisture Content, %	Screw Speed, rpm	Feed Rate, kg/h	Specific Mechanical Energy, SME, kJ/kg	Melt Temperature, °C
Native	-	-	-	-	-	-	-
LS-LT	Low SME-Low Temperature	80	30	200	13.2	74	83
LS-HT	Low SME-High Temperature	120	30	200	13.2	74	105
HS-LT	High SME-Low Temperature	80	17	200	13.2	145	100
HS-HT	High SME-High Temperature	120	17	200	13.2	145	126

**Table 2:** Effects of the modification of breadfruit flour with extrusion on flour proximate composition, pH, water holding capacity, and oil holding capacity.

	Extrusion Treatment ^1^					SEM ^2^	*p*-Value
	Native Flour	LS-LT	LS-HT	HS-LT	HS-HT		
Proximate composition							
As is basis							
Moisture, %	11.58 ^a^	6.57 ^d^	7.47 ^b^	6.98 ^c^	6.07 ^e^	0.06	<0.01
Starch, %	58.88 ^b^	67.44 ^a^	65.76 ^a,b^	63.85 ^a,b^	67.49 ^a^	1.64	0.02
Protein, %	4.91 ^b,c^	5.07 ^a,b^	4.87 ^c^	5.15 ^a^	5.09 ^a,b^	0.05	<0.01
Lipid, %	3.10 ^a^	1.86 ^b^	1.65 ^c^	1.80 ^b,c^	1.64 ^c^	0.04	<0.01
Ash, %	2.91 ^c^	3.18 ^a,b^	3.15 ^b^	3.22 ^a^	3.19 ^a,b^	0.01	<0.01
Dry matter basis							
Starch, %	66.59	72.19	71.06	68.64	71.85	1.76	0.20
Protein, %	5.54 ^a^	5.43 ^a,b^	5.26 ^b^	5.53 ^a^	5.41 ^a,b^	0.05	0.01
Lipid, %	3.51 ^a^	1.99 ^b^	1.78 ^b,c^	1.94 ^b,c^	1.74 ^c^	0.05	0.01
Ash, %	3.30 ^c^	3.40 ^b^	3.41 ^b^	3.46 ^a^	3.40 ^b^	0.01	<0.01
pH	5.74 ^d^	7.16 ^a^	6.99 ^b^	7.04 ^b^	6.73 ^c^	0.02	<0.01
Water holding capacity, g water/g flour	2.62 ^c^	4.89 ^a^	5.01 ^a^	2.99 ^b^	2.66 ^c^	0.06	<0.01
Oil holding capacity, g oil/g flour	2.02 ^a^	1.33 ^b^	1.39 ^b^	1.15 ^c^	1.40 ^b^	0.03	<0.01

^a–e^ Least square means lacking a common superscript letter within a row are different (*p* < 0.05). ^1^ Treatments include native breadfruit flour and four extruded breadfruit flours. Native breadfruit flour. LS-LT: SME = 74 kJ/kg, melt temperature = 83 °C. LS-HT: SME = 74 kJ/kg, melt temperature = 105 °C. HS-LT: SME = 145 kJ/kg, melt temperature = 100 °C. HS-HT: SME = 145 kJ/kg, melt temperature = 126 °C. ^2^ Maximum SEM (standard error of the mean) was reported.

**Table 3:** Effects of modification of breadfruit flour with extrusion on pasting properties of flours.

	Extrusion Treatment ^1^					SEM ^2^	*p*-Value
	Native Flour	LS-LT	LS-HT	HS-LT	HS-HT		
Cold peak viscosity, cP	ND ^3^	726.00 ^b^	1586.33 ^a^	679.67 ^b^	487.33 ^c^	19.08	<0.01
Hot peak viscosity, cP	2846.67 ^a^	978.67 ^b^	ND	ND	ND	24.28	<0.01
Breakdown, cP	496.00 ^b^	784.00 ^a^	ND	ND	ND	36.83	<0.01
Final Viscosity, cP	6850.00 ^a^	583.00 ^b^	542.00 ^b^	260.67 ^c^	222.33 ^c^	21.72	<0.01
Setback, cP	4499.33 ^a^	388.33 ^b^	378.00 ^b^	169.33 ^c^	144.67 ^c^	29.76	<0.01
Peak time, min	12.22 ^a^	9.49 ^b^	ND	ND	ND	0.04	<0.01

^a–c^ Least square means lacking a common superscript letter within a row are different (*p* < 0.05). ^1^ Treatments include native breadfruit flour and four extruded breadfruit flours. Native breadfruit flour. LS-LT: SME = 74 kJ/kg, melt temperature = 83 °C. LS-HT: SME = 74 kJ/kg, melt temperature = 105 °C. HS-LT: SME = 145 kJ/kg, melt temperature = 100 °C. HS-HT: SME = 145 kJ/kg, melt temperature = 126 °C. ^2^ Maximum SEM (standard error of the mean) was reported. ^3^ ND is not detected.

**Table 4:** Effects of the modification of breadfruit flour with extrusion on the cooking loss, texture (storage day 1), and instrumental color of soy protein isolated gels.

	Extrusion Treatment ^1^					SEM ^2^	*p*-Value
	Native Flour	LS-LT	LS-HT	HS-LT	HS-HT		
Cooking loss, %	1.36	1.21	1.32	1.33	1.40	0.06	0.29
Texture profile analysis							
Hardness, N	12.14 ^a^	9.58 ^b^	8.10 ^c^	5.82 ^d^	4.96 ^e^	0.12	<0.01
Gumminess, no units	9.62 ^a^	7.48 ^b^	6.20 ^c^	4.35 ^d^	3.63 ^e^	0.08	<0.01
Springiness, %	95.27 ^a^	94.65 ^a^^,^^b^	94.78 ^a,b^	93.80 ^b,c^	93.30 ^c^	0.31	<0.01
Adhesiveness g·sec	−0.55 ^a^	−1.18 ^b^	−1.06 ^b^	−0.95 ^b^	−0.99 ^b^	0.05	<0.01
Instrumental color							
L* (lightness)	63.73 ^a^	62.69 ^a^	62.84 ^a^	62.97 ^a^	60.87 ^b^	0.27	<0.01
a* (redness)	1.77 ^d^	2.28 ^b,c^	2.22 ^c^	2.34 ^b^	2.74 ^a^	0.03	<0.01
b* (yellowness)	14.72 ^a^	14.19 ^b^	14.71 ^a^	14.03 ^b^	14.66 ^a^	0.09	<0.01

^a–e^ Least square means lacking a common superscript letter within a row are different (*p* < 0.05). ^1^ Treatments include native breadfruit flour and four extruded breadfruit flours. Native breadfruit flour. LS-LT: SME = 74 kJ/kg, melt temperature = 83 °C. LS-HT: SME = 74 kJ/kg, melt temperature = 105 °C. HS-LT: SME = 145 kJ/kg, melt temperature = 100 °C. HS-HT: SME = 145 kJ/kg, melt temperature = 126 °C. ^2^ Maximum SEM (standard error of the mean) was reported.
